# High prevalence of tau pathologies in late-onset psychosis: A PET study

**DOI:** 10.1038/s41380-026-03749-3

**Published:** 2026-08-03

**Authors:** Manabu Kubota, Shin Kurose, Kenji Tagai, Yuki Momota, Masanori Ichihashi, Hironobu Endo, Chie Seki, Sho Moriguchi, Yasuharu Yamamoto, Yuko Kataoka, Ryoji Goto, Takehiro Tamura, Hiroki Shiwaku, Kiwamu Matsuoka, Hisaomi Suzuki, Mitsumoto Onaya, Takahiko Tokuda, Hiroyuki Uchida, Hidehiko Takahashi, Kazunori Kawamura, Ming-Rong Zhang, Makoto Higuchi, Keisuke Takahata

**Affiliations:** 1 Advanced Neuroimaging Center, Institute for Quantum Medical Science, National Institutes for Quantum Science and Technology, 4-9-1 Anagawa, Inage-ku, Chiba, Chiba 263-8555 Japan; 2 Department of Psychiatry, Kyoto University Graduate School of Medicine, 54 Shogoin Kawahara-cho, Sakyo-ku, Kyoto, Kyoto 606-8507 Japan; 3 Department of Neuropsychiatry, Keio University School of Medicine, 35 Shinanomachi, Shinjuku-ku, Tokyo, 160-8582 Japan; 4 Department of Psychiatry, Jikei University Graduate School of Medicine, 3-19-18 Nishi-Shinbashi, Minato-ku, Tokyo, 105-8461 Japan; 5 Department of Psychiatry and Behavioral Sciences, Institute of Science Tokyo, 1-5-45 Yushima, Bunkyo-ku, Tokyo, 113-8519 Japan; 6 Department of Psychiatry, Nara Medical University, 840 Shijo-cho, Kashihara, Nara, Nara 634-8522 Japan; 7 Department of Psychiatry, National Hospital Organization Shimofusa Psychiatric Center, 578 Hetacho, Midori-ku, Chiba, Chiba 266-0007 Japan; 8 Department of Advanced Nuclear Medicine Sciences, Institute for Quantum Medical Science, Quantum Life and Medical Science Directorate, National Institutes for Quantum Science and Technology, 4-9-1 Anagawa, Inage-ku, Chiba, Chiba 263-8555 Japan; 9 Neuroetiology and Diagnostic Science, Osaka Metropolitan University Graduate School of Medicine, 1-4-3 Asahi-machi, Abeno-ku, Osaka, Osaka 545-8585 Japan

**Keywords:** Neuroscience, Schizophrenia, Psychiatric disorders

## Abstract

Late-onset psychosis (LOP) exhibits distinct clinical features compared with younger-onset psychosis. Although postmortem and epidemiological studies have suggested an association between LOP and neurodegenerative processes, particularly tauopathies, in vivo evidence remains limited. This study aimed to investigate the involvement of Alzheimer’s disease (AD) and non-AD tauopathies in LOP using amyloid PET and tau PET with florzolotau (18F) (^18^F-florzolotau; also known as ^18^F-APN-1607 or ^18^F-PM-PBB3), a tracer capable of detecting a broad range of tau pathologies. Thirty-seven patients with LOP and 47 age-matched controls underwent PET scans with ^11^C-PiB and ^18^F-florzolotau to assess group differences in amyloid beta (Aβ) and tau accumulation. Diagnostic effects on regional ^18^F-florzolotau standardized uptake value ratios (SUVRs) were assessed. Associations between regional SUVRs and cognitive and clinical features were examined separately in Aβ-positive and Aβ-negative subgroups. Patients exhibited significantly higher positivity rates for both Aβ (13 of 37 patients, 35.1%) and tau (24 patients, 64.9%) than controls (one of 47 controls, 2.1% for Aβ and seven controls, 14.9% for tau). A significant diagnostic effect was observed on regional ^18^F-florzolotau SUVRs (*P* = 0.004), with post-hoc analyses revealing increased tracer retention in the parietal cortex. This diagnostic effect remained robust when the analysis was restricted to Aβ-negative participants (*P* = 0.002). Among Aβ-positive patients, greater parietal tau burden was associated with lower Frontal Assessment Battery scores. In conclusion, this in vivo PET study demonstrated a high prevalence of AD-like and non-AD-like tau accumulation patterns in LOP, suggesting that LOP may be associated with heterogeneous tau-related neurodegenerative processes.

## Introduction

Psychotic disorders, characterized by symptoms such as hallucinations and delusions, encompass a range of psychiatric conditions. Schizophrenia, the most studied form, typically manifests in adolescence or early adulthood. However, as early as 1943, Bleuler noted a subgroup of patients with late-onset presentations (onset after 40 years of age) [1]. Subsequent studies further clarified these cases as late-onset schizophrenia (LOS; onset between 40 and 60 years) and very-late-onset schizophrenia-like psychosis (VLOSLP; onset after 60 years), which exhibit clinical features distinct from early-onset schizophrenia [2]. Patients with LOS and VLOSLP often present with more prominent persecutory and partition delusions but fewer negative symptoms or formal thought disorders [2]. These clinical differences raise the possibility that late-onset psychosis (LOP) may involve distinct neurobiological mechanisms. Although conclusive evidence has yet to be established, small-scale neuroimaging studies have reported white matter (WM) hyperintensities, reduced WM integrity in posterior brain regions (e.g., the parietal lobe and posterior cingulum), and altered cerebral perfusion patterns in LOP [3–5], suggesting distinct neurobiological underpinnings compared with typical schizophrenia.

Supporting this hypothetical view, a large epidemiological cohort study involving 15,000 individuals with VLOSLP reported an elevated risk of developing dementia within 20 years of psychotic onset [6]. This finding suggests that LOP may either reflect a prodromal stage of neurodegenerative dementia or share overlapping pathophysiological processes [7]. Indeed, postmortem studies have demonstrated an increased prevalence of neurodegenerative pathologies, including argyrophilic grain disease (AGD), Lewy body disease (LBD), and corticobasal degeneration (CBD), among patients with LOS and late-onset delusional disorders [8]. Furthermore, a recent case series revealed various four-repeat (4R) tauopathies in patients with LOP, with AGD being the most common pathology, alongside features of progressive supranuclear palsy (PSP) and CBD [9]. Collectively, these findings implicate diverse neurodegenerative pathologies, particularly tauopathies, in LOP pathogenesis.

Despite these findings, the in vivo characterization of neuropathology in LOP and other aging-related psychiatric disorders remains limited, as most previous studies have relied on postmortem data. This knowledge gap is largely due to the lack of reliable neuroimaging methods capable of detecting a broad spectrum of tau aggregates, including both Alzheimer’s disease (AD)-type and non-AD-type tau pathologies in living individuals. For example, earlier PET studies using ^18^F-FDDNP reported higher tracer binding in late-life depression [10], but insufficient specificity of the radioligand for tau versus amyloid precluded a clear interpretation.

We previously identified elevated tau PET signals using the first-generation tracer ^11^C-PBB3 in patients with late-life depression, particularly those with psychotic manifestations [11]. Subsequently, we developed florzolotau (18F) (^18^F-florzolotau; also known as ^18^F-APN-1607 or ^18^F-PM-PBB3), a second-generation tau tracer that enables in vivo visualization of a broad spectrum of tauopathies—including AD-type, 3-repeat (3R), and 4R non-AD pathologies—with enhanced contrast [12–16]. Notably, our recent study utilizing ^18^F-florzolotau revealed a high prevalence of tau positivity in patients with late-onset mood disorders, especially those with psychotic symptoms [17].

Building on these findings, the present study aimed to characterize the neuropathology underlying LOP by combining amyloid PET with ^11^C-PiB and tau PET with ^18^F-florzolotau. A particular focus was placed on characterizing the diverse patterns of tau accumulation associated with LOP. We hypothesized that amyloid and tau accumulation would be elevated in patients with LOP.

## Materials and methods

### Participants

This case-control study included 37 patients with LOP and 47 control participants. Statistical power analysis was performed using G*Power 3.1 software [18] for the estimation of sample size, and the effect size of our previous study involving amyloid beta (Aβ) and tau PET tracers [11] was 0.93. With this effect size, an alpha level of 0.01, and a power of 0.95, the required sample size for each group was calculated to be 21. All patients were recruited from the affiliated hospitals and clinics. The inclusion criteria for the patients were as follows:

1. clinical diagnosis of schizophrenia, schizoaffective disorder, delusional disorder, acute transient psychotic episode, or major depressive disorder with psychotic symptoms (i.e., psychotic depression), based on the Structural Clinical Interview for DSM-5
2. onset of the illness after the age of 40
3. no evidence of cognitive impairment or neurological diseases at illness onset, based on clinical records and interviews with family members or caregivers

The exclusion criteria included a history of severe traumatic brain injury, substance abuse, or any psychiatric disorder before the age of 40 years. One patient with psychotic depression in this study was also included in our previously published paper (Kurose et al. [17]) through the use of secondary data. This patient was enrolled in the present study prior to the initiation of Kurose et al.’s research in March 2020. No other patients overlapped between the two studies.

Cognitively unimpaired control participants were recruited by the National Institutes for Quantum Science and Technology as part of an ongoing study on late-life psychiatric disorders, as described previously [17]. All controls underwent psychiatric evaluation based on the DSM-5 criteria and had no history of neuropsychiatric or neurological disorders, severe medical illness, or substance abuse that could affect brain function. Participants with Mini-Mental State Examination (MMSE) scores < 28 were excluded. At the time of PET scanning, all patients and controls were physically healthy and had no neurological abnormalities.

All participants were clinically evaluated by at least two board-certified psychiatrists and one board-certified neurologist. Cognitive function was assessed using the MMSE and Frontal Assessment Battery (FAB). Psychopathological symptoms in patients were evaluated using either the Positive and Negative Syndrome Scale (PANSS) [19] or the Brief Psychiatric Rating Scale (BPRS) [20]. Recruitment and data collection were conducted between July 2017 and October 2023. The demographic and clinical characteristics of the participants are presented in Table 1.

**Table 1:** Characteristics of participants included in this study.

	Patients (N = 37)		Controls (N = 47)		Statistics	
	n	Mean (SD)	n	Mean (SD)	*T/chi-square*	*P*
Age, mean (SD), y	37	69.9 (9.0)	47	66.4 (8.7)	1.8	0.08
Sex (male/female)	37	10/27	47	25/22	5.8	0.016 ^4)^
Diagnosis						
Total		N = 37				
Schizophrenia		n = 20				
Schizoaffective disorder		n = 1				
Delusional disorder		n = 13				
Acute transient psychotic episode		n = 2				
Psychotic depression		n = 1				
Education level, mean (SD), y	37	11.9 (3.3)	47	14.6 (1.9)	−4.4	<0.001 ^4)^
MMSE, mean (SD) ^1)^	36	25.1 (3.8)	47	29.2 (0.8)	−6.4	<0.001 ^4)^
FAB, mean (SD) ^2)^	34	12.7 (3.6)	46	16.3 (1.3)	−5.6	<0.001 ^4)^
Duration of illness, mean (SD), y	37	5.7 (6.5)				
Age at onset, mean (SD), y	37	64.2 (11.1)				
Age at onset, y [40–60] / [>60]	37	13/24				
Partition delusion (yes/no)	37	13/24				
Persecutory delusion (yes/no)	37	30/7				
Hallucination (yes/no)	37	20/17				
Visual hallucination (yes/no)	37	0/37				
BPRS, mean (SD) ^3)^	20	33.6 (8.8)				
PANSS total, mean (SD) ^3)^	14	58.5 (22.4)				
PANSS positive, mean (SD) ^3)^	14	13.1 (6.2)				
PANSS negative, mean (SD) ^3)^	14	15.9 (6.6)				
PANSS general, mean (SD) ^3)^	14	29.4 (11.9)				
Converted BPRS, mean (SD) ^3)^	34	33.3 (10.2)				
Injected radioactivity for ^11^C-PiB, mean (SD), MBq	37	541.8 (22.0)	47	530.8 (53.0)	1.3	0.20
Injected radioactivity for ^18^F-florzolotau, mean (SD), MBq	37	186.7 (8.3)	47	187.3 (6.6)	−0.3	0.75

*BPRS*, Brief Psychiatric Rating Scale; *FAB*, Frontal Assessment Battery; *MMSE*, Mini‐Mental State Examination; *PANSS*, Positive and Negative Syndrome Scale; *SUVR*, standardized uptake value ratio.

1) Data are unavailable for one patient.

2) Data are unavailable for three patients and one control.

3) Clinical assessments of patients were conducted using either the BPRS or PANSS. Data for three patients are unavailable. PANSS total scores were converted to BPRS total score according to a previously validated method [29].

4) *P* < 0.05.

This study was approved by the Radiation Drug Safety Committee and the Institutional Review Board of the National Institutes for Quantum Science and Technology, Chiba, Japan. All procedures were conducted in accordance with the Code of Ethics of the World Medical Association. Written informed consent was obtained from all participants after a complete explanation of the study.

### Imaging data acquisition and processing

Details of the MRI and PET data acquisition and image reconstruction are provided in Supplementary Methods S1 and S2. Each participant underwent PET imaging with ^11^C-PiB and ^18^F-florzolotau to quantify Aβ and tau accumulation, respectively. Radiosynthesis of these PET tracers was performed as previously described [12, 21].

The motion-corrected PET images were co-registered to the individual T1-weighted MR images. Regions of interest (ROIs) were defined on MR images using standard anatomical atlases [22, 23]. Voxel-wise parametric images were generated for quantification. ^11^C-PiB standardized uptake value ratio (SUVR) maps were generated using cerebellar gray matter (GM) as reference region during the 50–70 min post-injection period. ^18^F-florzolotau SUVR maps were generated based on the optimized reference tissue method during the 90–110 min post-injection period, as previously described [24].

Details of the ROI definition process, an evaluation of potential spillover effects from ^18^F-florzolotau accumulation in the choroid plexus, and methodological considerations concerning the optimized reference tissue method are provided in Supplementary Methods S3, Supplementary Discussion S1–S2, and Supplementary Figure S1.

### Data analysis

#### Assessments of Aβ positivity

Aβ positivity was assessed using ^11^C-PiB PET images. Three independent raters classified each participant as Aβ-positive or Aβ-negative based on the standardized visual assessment procedures established by the Japanese Alzheimer’s Disease Neuroimaging Initiative [25].

#### Assessments of ^18^F-florzolotau PET data

Assessments of potential tau pathologies in patients were conducted by combining ROI-based and voxel-based approaches [17] as follows.

To investigate regional tau accumulation, cortical GM regions in the frontal, temporal, parietal, and occipital lobes were defined by merging the corresponding ROIs (see Supplementary Methods S3 for details). A multivariate analysis of covariance (MANCOVA) was conducted to evaluate the effects of diagnosis (patients = 1, controls = 0) on ^18^F-florzolotau SUVR across the four regions. SUVR values served as dependent variables, while age and sex were included as covariates. A statistical threshold of *P* < 0.05 was used. When the main effect of diagnosis was significant, post-hoc analyses were performed to assess the SUVR differences in each ROI, with the statistical threshold set at *P* < 0.05 with Bonferroni correction for the four ROIs (*P* < 0.05/4).

Subsequently, the same MANCOVA was applied to Aβ-negative participants. Additionally, within the Aβ-negative patient group, MANCOVA was conducted to compare SUVRs between the following two subgroups: those with symptom onset between 40 and 60 years of age, and those with onset after 60 years of age, controlling for age and sex.

Lastly, we assessed individual-level tau positivity and spatial tau distribution patterns by voxel-wise analysis of ^18^F-florzolotau SUVR images [17], as described in Supplementary Methods S4. Group differences in the proportion of tau-positive individuals were examined using the chi-square test. A *P*-value of < 0.05 was considered statistically significant.

In light of previous evidence suggesting that educational attainment may influence amyloid and tau burden [26–28], supplementary analyses were conducted to examine whether our findings remained significant after controlling for educational level (Supplementary Methods S5 and Supplementary Results S1). Furthermore, to address the disparities in the sex ratio and age distribution between the LOP and control groups, sensitivity analyses were performed by excluding healthy male controls younger than 65 years (Supplementary Methods S6, Supplementary Results S2, and Supplementary Table S1).

#### Associations between ^18^F-florzolotau accumulation and cognitive and clinical characteristics

To explore the relationship between tau accumulation and cognitive and clinical features, correlational analyses were conducted separately for the Aβ-positive and Aβ-negative patients. For each group, ^18^F-florzolotau SUVR values in the frontal, temporal, parietal, and occipital ROIs were correlated with the following variables: a) MMSE total score, b) FAB total score, and c) overall clinical symptom severity. To ensure compatibility in the assessment of clinical symptom severity, PANSS total scores (available in 14 patients) were converted to equivalent BPRS total scores using a previously validated method [29]. These converted scores were analyzed alongside original BPRS scores from the remaining patients.

Spearman’s rank correlation coefficients were used for MMSE scores due to their non-normal distribution, while Pearson’s correlation coefficients were applied for FAB scores and symptom severity. Given the exploratory nature of these analyses, statistical significance was set at *P* < 0.05.

Additional analyses examining the association between ^18^F-florzolotau SUVRs and the presence or history of clinical symptoms related to psychosis were performed, as described in Supplementary Methods S7.

## Results

### Demographic characteristics

The demographic characteristics of the participants are presented in Table 1.

Compared to the control group, patients with LOP showed significantly lower MMSE and FAB scores and educational attainment. Symptom onset occurred after the age of 60 years in 24 individuals, whereas the remaining 13 experienced onset between 40 and 60 years of age.

### Aβ positivity assessed by ^11^C-PiB-PET

Aβ depositions were detected in 13 of the 37 patients (35.1%) and in one of the 47 control participants (2.1%). The proportion of Aβ positivity was significantly higher in the patient group than in the control group (*χ*^*2*^ = 16.2, *P* < 0.001, Cramer’s *V* = 0.44). Notably, all Aβ-positive patients had psychosis onset after the age of 60 years.

### Group comparison of ^18^F-florzolotau retention

**Fig. 1:**
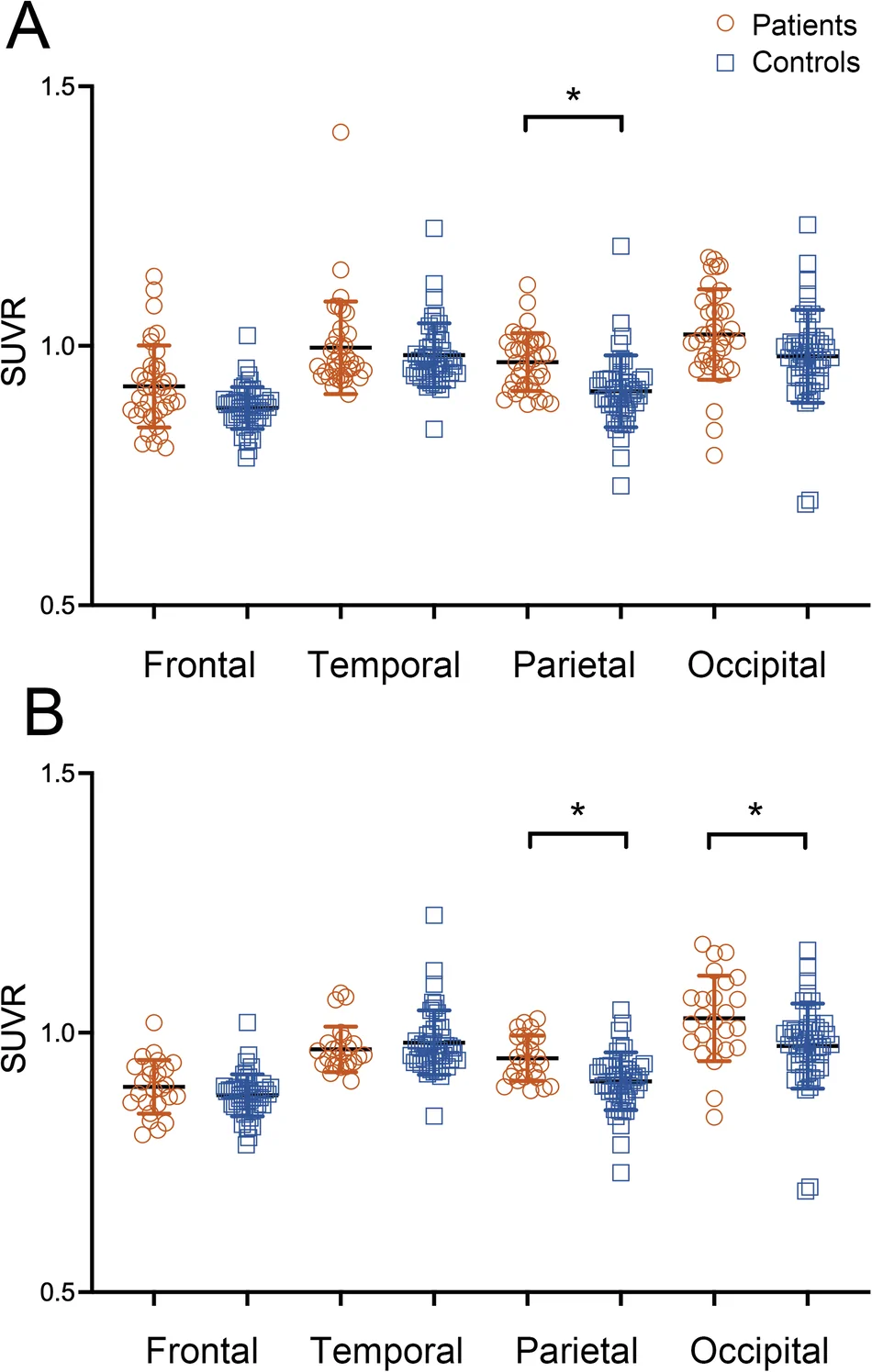
Comparisons of ^18^F-florzolotau SUVR in each region between patients and controls. (**A**) All participants (patients, n = 37; controls, n = 47); (**B**) Aβ-negative participants only (patients, n = 24; controls, n = 46). Error bars represent the standard deviation. Aβ, amyloid-beta; SUVR, standardized uptake value ratio. * *P* < 0.05/4.

**Fig. 2:**
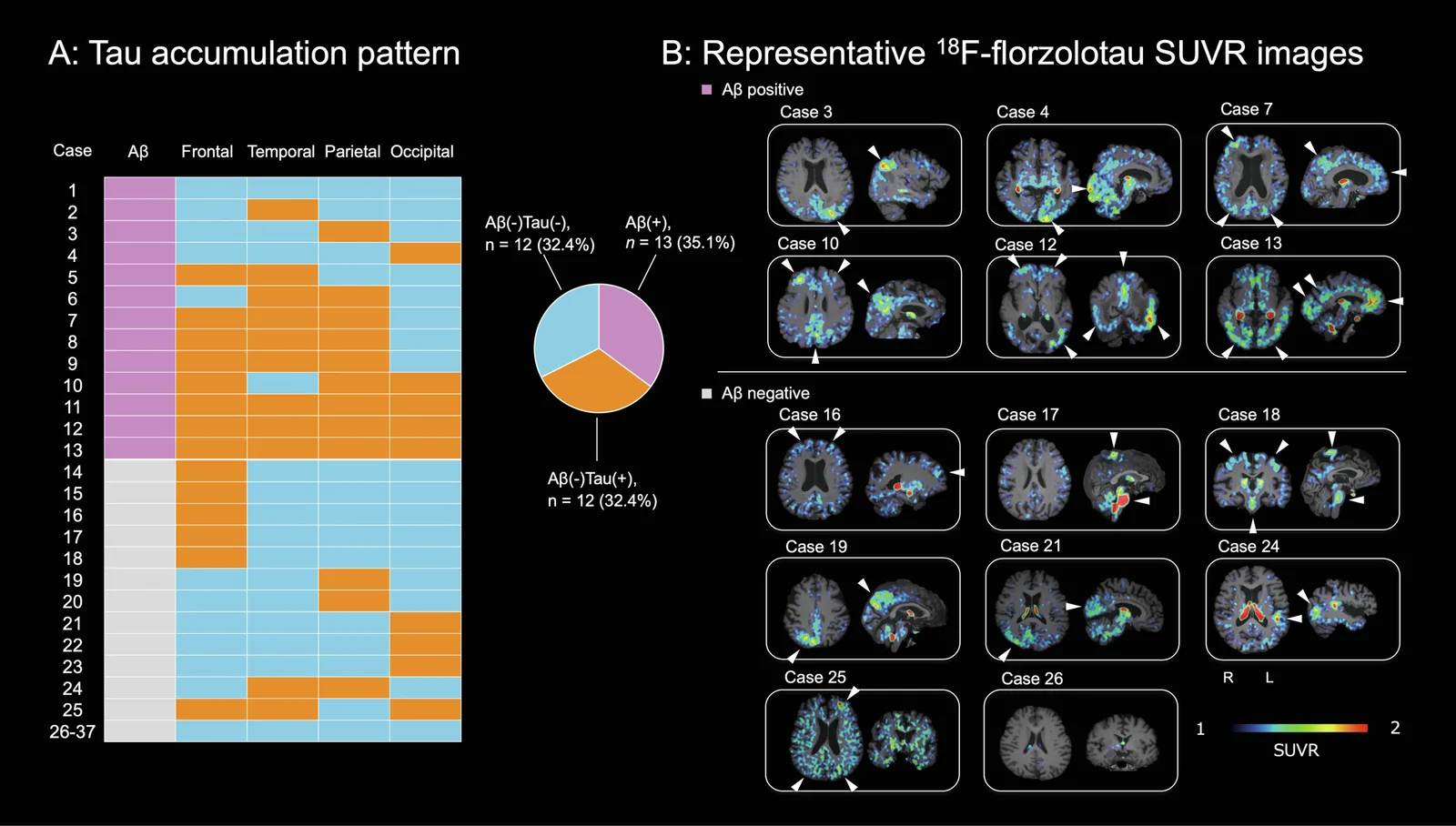
Topological tau accumulation pattern and representative ^18^F-florzolotau PET images in patients. (**A**) Topological patterns of tau accumulation assessed individually using ^18^F-florzolotau SUVR data, along with positivity for Aβ and tau in patients. Cases 1–13 were identified as Aβ-positive, as denoted by the purple color in the Aβ column of this figure. Cases 14–25 were Aβ-negative and tau-positive, while cases 26–37 were both Aβ-negative and tau-negative. Regions classified as tau-positive and tau-negative are displayed in orange and light blue, respectively. (**B**) Representative ^18^F-florzolotau SUVR images in the patient group. White arrowheads indicate characteristic regional patterns of tracer retention, including unilateral parietal or occipital predominance (Cases 3, 4, 19, 21), concurrent frontal and posterior cortical involvement with varying degrees of diffuse accumulation (Cases 7, 10, 12, 13, 16, 25), precentral gyrus and brainstem accumulation (Cases 17, 18), and asymmetrical temporoparietal accumulation (Case 24). Aβ, amyloid-beta; SUVR, standardized uptake value ratio.

MANCOVA revealed a significant main effect of diagnosis on ^18^F-florzolotau SUVRs (*P* = 0.004). Post-hoc univariate analyses showed significantly higher SUVRs in the parietal ROI of patients than in controls (*P* < 0.001), along with trend-level increases in the frontal (*P* = 0.013) and occipital (*P* = 0.019) ROIs (Table 2A; Figs. 1A and 2). The elevated parietal SUVRs in the patient group remained statistically significant even after exclusion of the amyloid-positive patient with psychotic depression (*P* = 0.001).

**Table 2:** Group differences in ^18^F-florzolotau SUVR between patients and controls.

(A) All participants									
	Patients (n = 37)	Controls (n = 47)	Multivariate tests (effect of diagnosis)			Between-subjects effects			
SUVR	Mean (SD)	Mean (SD)	*F*(4,63)	*P*	Partial η^2^/Power	*F*(1,66)	*P*	Partial η^2^	Power
			4.13	0.004 ^1)^	0.18/0.90				
Frontal	0.92 (0.08)	0.88 (0.04)				6.47	0.013	0.07	0.71
Temporal	1.00 (0.09)	0.98 (0.06)				0.26	0.61	0.003	0.08
Parietal	0.97 (0.06)	0.91 (0.07)				12.17	0.001 ^2)^	0.13	0.93
Occipital	1.02 (0.09)	0.98 (0.09)				5.76	0.019	0.07	0.66

(B) Participants with amyloid negativity only									
	Patients (n = 24)	Controls (n = 46)	Multivariate tests (effect of diagnosis)			Between-subjects effects			
SUVR	Mean (SD)	Mean (SD)	*F*(4,63)	*P*	Partial η^2^/Power	*F*(1,66)	*P*	Partial η^2^	Power
			4.77	0.002 ^1)^	0.23/0.94				
Frontal	0.90 (0.05)	0.88 (0.04)				1.41	0.24	0.02	0.22
Temporal	0.97 (0.04)	0.98 (0.06)				1.49	0.23	0.02	0.23
Parietal	0.95 (0.04)	0.91 (0.06)				8.69	0.004 ^2)^	0.12	0.83
Occipital	1.03 (0.08)	0.97 (0.08)				6.77	0.011 ^2)^	0.09	0.73

*SUVR*, standardized uptake value ratio.

1) *P* < 0.05 (effect of diagnosis).

2) *P* < 0.05/4 (post-hoc between-subjects effects).

When the analysis was restricted to Aβ-negative participants, the diagnostic effect on SUVRs remained significant (MANCOVA, *P* = 0.002). Follow-up univariate analyses revealed significantly higher SUVRs in the parietal (*P* = 0.004) and occipital (*P* = 0.011) ROIs in Aβ-negative patients than in Aβ-negative controls (Table 2B; Figs. 1B and 2).

Among Aβ-negative patients, subgroup analysis compared individuals with psychosis onset between the ages of 40 and 60 years and those with onset after 60 years. No significant main effect of the subgroup was found, indicating that the age of onset within the late-onset spectrum does not significantly influence the degree of tau accumulation.

At the individual level, voxel-wise analysis of ^18^F-florzolotau SUVR images identified tau positivity in 12 of 13 Aβ-positive patients and in 12 of 24 Aβ-negative patients. These tau-positive patients exhibited topologically heterogeneous distribution patterns of tracer retention (Fig. 2). Among the Aβ-positive patients, the tau deposition patterns included unilateral parietal or occipital predominance (Cases 3 and 4), concurrent involvement of the frontal and posterior cortical regions (Cases 10 and 12), and varying degrees of widespread accumulation with frontal and parietal predominance (Cases 7 and 13) (Fig. 2B). These distribution patterns were broadly consistent with tau pathology corresponding to different Braak tau stages. In contrast, the Aβ-negative, tau-positive patients demonstrated greater heterogeneity, including unilateral parietal or occipital predominance (Cases 19 and 21), localized frontal accumulation with varying degrees of diffuse cortical involvement (Cases 16 and 25), concurrent involvement of the precentral gyrus and brainstem (Cases 17 and 18), and asymmetrical temporoparietal deposition (Case 24) (Fig. 2B). On the other hand, seven of the 47 control participants were classified as tau-positive, as reported in our previous study [17]. Overall, tau deposition was detected in 24 (64.9%) patients and seven (14.9%) controls. The proportion of tau positivity was significantly higher in the patient group than in the control group (*χ*^*2*^ = 22.2, *P* < 0.001, Cramer’s *V* = 0.51).

### Associations between ^18^F-florzolotau accumulation and cognitive and clinical characteristics

**Fig. 3:**
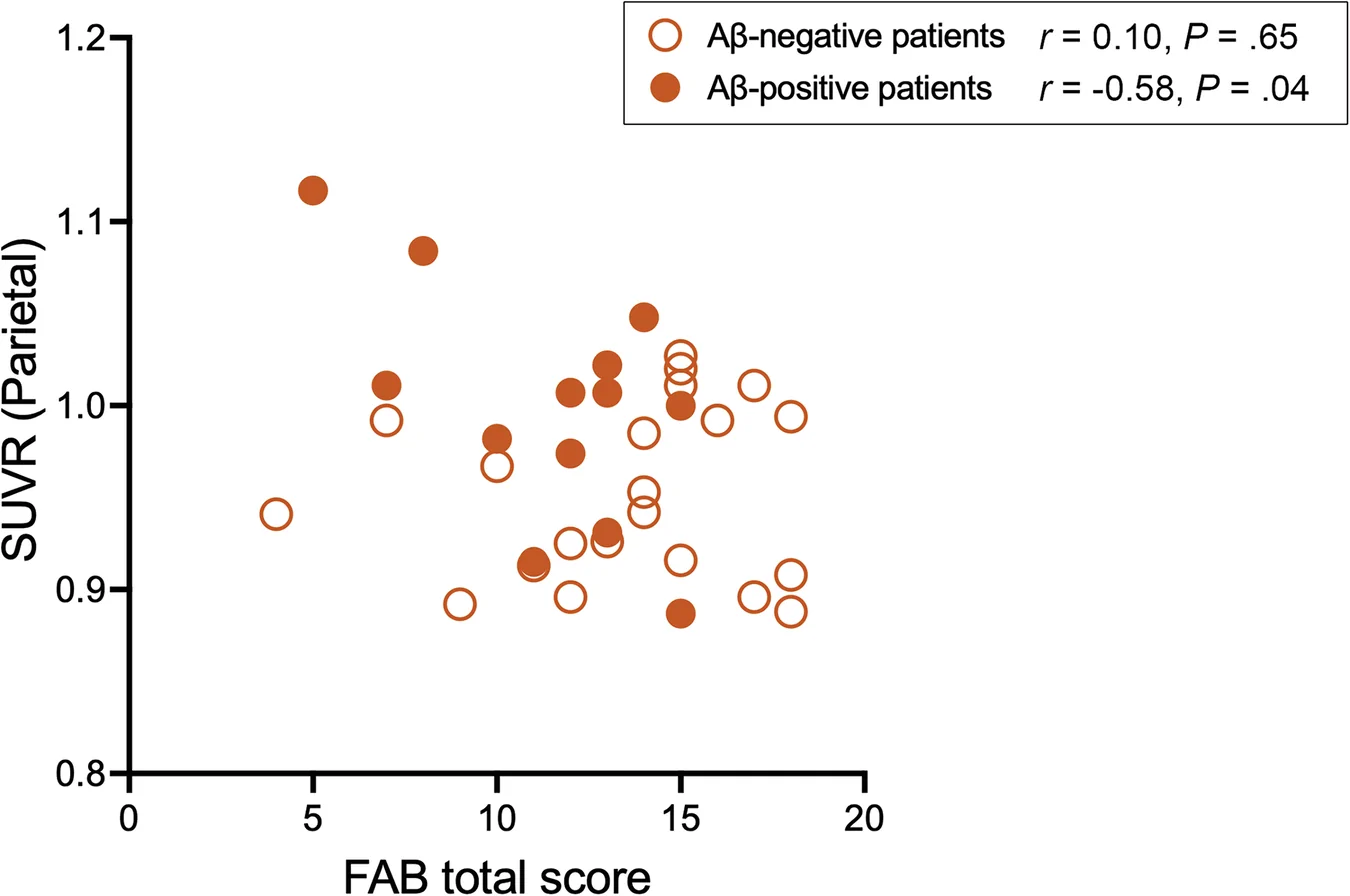
Scatter plot of ^18^F-florzolotau SUVR in the parietal region against FAB total scores in patients. Filled circles indicate patients who are Aβ-positive. Aβ-negative patients, n = 21; Aβ-positive patients, n = 13. Aβ, amyloid-beta; FAB, Frontal Assessment Battery; *r*, Pearson’s *r* ; SUVR, standardized uptake value ratio.

In Aβ-positive patients, a significant negative correlation was observed between the ^18^F-florzolotau SUVR in the parietal ROI and total FAB scores (Pearson’s *r* = −0.58, *P* = 0.04; Fig. 3), indicating that greater tau accumulation in this region is associated with more severe frontal executive functions. No other significant correlations between the SUVRs and cognitive or clinical measures were found in either Aβ-positive or Aβ-negative patients.

The presence or history of psychosis-related clinical symptoms showed no significant association with SUVRs in either the total patient cohort or the Aβ-negative subgroup (Supplementary Results S3).

## Discussion

In this study, we applied PET imaging with ^11^C-PiB and ^18^F-florzolotau to examine Aβ and tau pathologies in patients with LOP. We found a higher prevalence of amyloid positivity in the LOP group than in the control group. Importantly, the increase in tau accumulation was even more pronounced than that of Aβ deposition, and this elevated frequency of tau pathology persisted among Aβ-negative patients. These findings suggest that both AD-related and non-AD tau pathologies contribute to the etiology of LOP.

The 37 patients with LOP consisted of three subgroups: those with AD-type pathology (n = 13; 35.1%), those with non-AD tauopathy (n = 12; 32.4%), and those with non-AD/non-tau condition (n = 12; 32.4%), with the proportions being nearly equal (Fig. 2). There has been no clear in vivo evidence and limited neuropathological information supporting the association of Aβ and tau depositions with LOP. As summarized in a systematic review of five postmortem studies [30], LOP cases exhibit marked heterogeneity in the frequency and type of neurodegenerative pathologies, ranging from no excess of Aβ or tau deposits compared with controls to an increased prevalence of non-AD proteinopathies [8, 31, 32]. Such discrepancies may reflect differences in the settings and methods of longitudinal follow-up, leading to autopsies. A more recent study of patients with VLOSLP with a mean age in the early 80 s reported Aβ positivity in 9 of 17 cases [33]. In our cohort, all 13 Aβ-positive patients had disease onset after the age of 60 years, corresponding to 13 out of 24 patients in this age subgroup, consistent with the VLOSLP findings.

Although patients as a group exhibited prominent ^18^F-florzolotau accumulation in the parietal cortex, Aβ-positive individuals showed diverse tau deposition patterns, including posterior cortical predominance, asymmetrical or symmetrical temporoparietal predominance, and marked frontal involvement accompanied by posterior cortical accumulation (Fig. 2). These patterns align with the tau distributions described in AD subtypes [34, 35], suggesting that multiple AD-related tau phenotypes may underlie LOP. Furthermore, the high rate of amyloid positivity observed in our cohort may partly reflect a co-pathology of AD and LBD [36] in some cases, as these conditions frequently co-occur [37]. Clarification of this possibility requires further investigation using PET tracers for α-synuclein [38, 39].

The parietal tau accumulation observed in the total and Aβ-negative LOP cases appears to be distinct from the augmented frontal tau depositions in late-onset mood disorders with psychotic symptoms [17]. Although the current LOP cohort included one patient with psychotic depression who was amyloid-positive and also included in our previously published study [17], the predominant parietal tau distribution remained consistent even after excluding this individual. Both the parietal and frontal regions are core components of the central executive network (also referred to as the frontoparietal network), which is essential for allocating attentional resources and directing top-down control [40]. This network has also been critically implicated in the pathophysiology of early and chronic psychotic symptoms [41]. Our findings suggest that while frontal dysfunction may be more closely associated with mood-related psychosis [17], parietal-dominant tau pathology could reflect a distinct neurodegenerative process contributing to the emergence of clinical symptoms in LOP, potentially through dysfunction of the posterior nodes of the central executive network.

Besides contributing to psychotic features, disruptions of the frontoparietal network could also provoke cognitive deficits, as greater tau accumulation in the parietal cortex of Aβ-positive LOP patients was correlated with lower FAB scores. This observation is in line with recent findings in AD-spectrum cases, where tau deposition has been linked to impaired executive functions through the deterioration of the frontotemporal network [42]. Nevertheless, the possibility remains that disturbances in other networks connected to regions of tau accumulation additionally led to declines in FAB performance, as suggested by our recent lesion network mapping analysis of tau PET images in patients with PSP [43].

It is also noteworthy that half of the Aβ-negative individuals with LOP showed tau positivity (Fig. 2), with considerable variability in tau distributions as outlined in the Results. These topological patterns included parietal and frontal involvements, unilateral predominance in the parietal or occipital cortex, precentral and brainstem depositions, and parietal-occipital involvement with focal frontal accumulation. Given the ability of ^18^F-florzolotau to detect non-AD tau pathologies, including 3R and 4R aggregates [12, 14], these findings suggest a heterogeneous neuropathological background potentially comprising diverse primary tauopathies. For instance, tau accumulation in the precentral cortex with concurrent brainstem involvement may reflect a distribution characteristic of 4R tauopathy, although imaging-pathology correlation studies are required to substantiate this interpretation. Notably, no significant associations were found between ^18^F-florzolotau SUVR in any specific region and cognitive or clinical measures or the presence of current or past psychotic symptoms in Aβ-negative LOP cases, implying the diversity of underlying pathologies. Thus, key cognitive and psychotic manifestations may arise from a shared neural network integrating multiple tau-affected regions, which could be delineated by lesion network mapping using a normative connectome database [43].

The observed elevations in Aβ and tau positivity rates among patients with LOP suggest that this condition may represent a prodromal stage of neurodegenerative dementias characterized by cerebral proteinopathies. Consistently, postmortem studies of LOS and delusional disorders have demonstrated associations with diverse pathologies, including CBD, LBD, AGD, and primary age-related tauopathy [8, 9, 32]. Other clinicopathological investigations have further revealed links between psychosis and mixed LBD/AD pathology, as well as TDP-43 pathology [37]. In clinical settings, psychosis is also observed in patients with prodromal AD (i.e., early-stage mild cognitive impairment) and dementia with Lewy bodies [44]. Notably, however, none of our patients had visual hallucinations, a psychotic symptom commonly observed in dementia with Lewy bodies and its prodromal stages. Longitudinal clinical and PET studies will allow the pursuit of the transition from LOP to dementing illnesses with different neuropathological phenotypes, thereby informing the timing of disease-modifying interventions prior to the onset of overt cognitive deficits.

Our supplementary analyses confirmed that the elevated prevalence of Aβ and tau positivity, as well as the increased regional tau accumulation observed in the LOP group, remained significant after adjusting for educational attainment. However, in Aβ-negative patients, the increase in occipital ^18^F-florzolotau SUVR was attenuated from statistical significance to a trend-level effect after controlling for education. As educational attainment was significantly lower in patients than in controls, this finding raises the possibility that lower premorbid educational levels may partially contribute to enhanced tau accumulation in Aβ-negative LOP. This interpretation is consistent with previous studies in aging populations, suggesting that lower educational attainment is associated with a greater tau burden in the absence of amyloid pathology [45]. Nevertheless, given that the primary findings remained significant after adjusting for educational attainment, the elevated tau burden in LOP is unlikely to be predominantly explained by differences in educational background.

Regarding the potential influence of age and sex on our findings, sensitivity analyses excluding healthy male controls younger than 65 years of age yielded results that were largely consistent with the primary findings. Although the increase in occipital ^18^F-florzolotau accumulation in Aβ-negative patients reached only trend-level significance, possibly due to the reduced sample size, the overall findings support the robustness and general consistency of the observed proteinopathy characteristics in LOP.

In Aβ-negative patients, no significant difference in tau accumulation was detected between individuals with disease onset at or before 60 years of age and those with disease onset after 60 years. Although previous studies have suggested a link between VLOSLP and neurodegenerative changes, as well as the potential early manifestation of underlying dementia [6, 8], our findings did not elucidate whether the high prevalence of non-AD tauopathy is specific to VLOSLP among LOP and contributes to this reported association. Nevertheless, given the relatively small sample size, further research involving larger cohorts is necessary to determine whether pathophysiological differences exist between these age-defined subgroups or between LOS and VLOSLP.

This study has several limitations. First, the severity of psychotic symptoms was evaluated using either BPRS and PANSS. This mixed use may have influenced the analysis of correlations between tau deposition and psychiatric measures, although we applied a validated method to convert PANSS total scores into BPRS equivalents [29]. Second, no postmortem neuropathological data were acquired from the patients included in this study, underscoring the need for future studies to validate the accuracy of the current imaging findings. Finally, the sample size of our patient group is relatively small, which may limit the overall statistical power and the generalizability of the findings. Therefore, while the current findings, including those from the subgroup analyses, provide informative insights into the potential neurobiological mechanisms of LOP, they should be interpreted as exploratory. These findings warrant further validation in larger, independent cohorts using multi-center designs to confirm their robustness.

In conclusion, this study provides novel in vivo evidence suggesting that LOP may involve heterogeneous cerebral proteinopathies, as reflected by the ability of ^18^F-florzolotau PET to detect diverse tau pathologies. This neuroimaging approach further implies a potential contribution of tau-related pathology to network dysfunction underlying cognitive impairment. Collectively, these findings highlight the potential significance of LOP as a clinically important interface between psychiatric and neurodegenerative disorders and possibly as a prodromal stage of multiple dementing disorders.

## Supplementary information

Supplementary Information

## Data Availability

The datasets analyzed during the current study are not publicly available due to concerns regarding participant privacy and consent, but are available from the corresponding author upon reasonable request.
