# Supplementary material for: High prevalence of tau pathologies in late-onset psychosis: A PET study
Source: Mol Psychiatry. 2026 Aug 3;31(12):6881–8. doi: 10.1038/s41380-026-03749-3 (PMC13736494; doi:10.1038/s41380-026-03749-3)
Supplement: Supplementary file 1 [file 41380_2026_3749_MOESM1_ESM.docx]

**Supplementary Information**

**Title:**

**High prevalence of tau pathologies in late-onset psychosis: A PET study**

**Authors:**

Manabu Kubota M.D., Ph.D.^1,2,10^, Shin Kurose M.D., Ph.D.^1,3,10^, Kenji Tagai M.D., Ph.D.^1,4^, Yuki Momota Ph.D.^1^, Masanori Ichihashi M.D.^1,5^, Hironobu Endo M.D., Ph.D.^1^, Chie Seki Ph.D.^1^, Sho Moriguchi M.D., Ph.D.^1,3^, Yasuharu Yamamoto M.D., Ph.D.^1,3^, Yuko Kataoka M.D., Ph.D.^1^, Ryoji Goto M.D., Ph.D.^1^, Takehiro Tamura M.D., Ph.D.^5^, Hiroki Shiwaku M.D., Ph.D.^5^, Kiwamu Matsuoka M.D., Ph.D.^1,6^, Hisaomi Suzuki M.D., Ph.D.^1,7^, Mitsumoto Onaya M.D., Ph.D.^7^, Takahiko Tokuda M.D., Ph.D.^1^, Hiroyuki Uchida M.D., Ph.D.^3^, Hidehiko Takahashi M.D., Ph.D.^5^, Kazunori Kawamura Ph.D.^8^, Ming-Rong Zhang Ph.D.^8^, Makoto Higuchi M.D., Ph.D.^1,9^, Keisuke Takahata M.D., Ph.D.^1,3^

**Affiliations:**

^1^ Advanced Neuroimaging Center, Institute for Quantum Medical Science, National Institutes for Quantum Science and Technology, 4-9-1 Anagawa, Inage, Chiba, Chiba 263-8555, Japan

^2^ Department of Psychiatry, Kyoto University Graduate School of Medicine, 54 Shogoin Kawahara-cho, Sakyo-ku Kyoto 606-8507, Japan

^3^ Department of Neuropsychiatry, Keio University School of Medicine, 35 Shinanomachi, Shinjuku, Tokyo 160-8582, Japan

^4^ Department of Psychiatry, Jikei University Graduate School of Medicine, Tokyo 105-8461, Japan

^5^ Department of Psychiatry and Behavioral Sciences, Institute of Science Tokyo, 1-5-45 Yushima, Bunkyo-ku, Tokyo, 113-8519, Japan

^6^ Department of Psychiatry, Nara Medical University, Nara 634-8521, Japan

^7^ Department of Psychiatry, National Hospital Organization Shimofusa Psychiatric Center, Chiba 266-0007, Japan

^8^ Department of Advanced Nuclear Medicine Sciences, Institute for Quantum Medical Science, Quantum Life and Medical Science Directorate, National Institutes for Quantum Science and Technology, 4-9-1 Anagawa, Inage, Chiba, Chiba 263-8555, Japan

^9^ Neuroetiology and Diagnostic Science, Osaka Metropolitan University Graduate School of Medicine, 1-4-3 Asahi-machi, Abeno-ku, Osaka 545-8585, Japan

^10^ These authors contributed equally to this work.

**Corresponding author:**

Manabu Kubota, M.D., Ph.D.

Advanced Neuroimaging Center, Institute for Quantum Medical Science, National Institutes for Quantum Science and Technology

Address: 4-9-1 Anagawa, Inage-ku, Chiba, Chiba 263-8555, Japan

Tel. +81-43-206-3251 Fax. +81-43-253-0396

Email: [kubota.manabu@qst.go.jp,](mailto:kubota.manabu@qst.go.jp) [m_kubota@kuhp.kyoto-u.ac.jp](mailto:m_kubota@kuhp.kyoto-u.ac.jp)

**Supplementary Methods S1.** MRI data acquisition and scan parameters, and data processing

**Supplementary Methods S2.** Details of PET scanners and acquisition protocols

**Supplementary Methods S3.** Details of ROI definition

**Supplementary Methods S4.** Individual evaluation of ^18^F-florzolotau images and tau positivity assessments in patients

**Supplementary Methods S5.** Group differences in amyloid and tau accumulation after adjustment for educational attainment

**Supplementary Methods S6.** Sensitivity analysis excluding healthy male controls younger than 65 years

**Supplementary Methods S7.** Relationship between ^18^F-florzolotau SUVR and the presence or history of clinical features related to psychosis

**Supplementary Results S1.** Analysis of group differences in amyloid and tau accumulation after adjustment for educational attainment

**Supplementary Results S2.** Sensitivity analysis excluding healthy male controls younger than 65 years

**Supplementary Results S3.** Relationship between ^18^F-florzolotau SUVR and the presence or history of clinical features related to psychosis

**Supplementary Discussion S1.** Potential spillover effects from ^18^F-florzolotau accumulation in the choroid plexus

**Supplementary Discussion S2.** Methodological considerations regarding the optimized reference tissue method

**Supplementary Table S1.** Group comparisons after excluding healthy male controls younger than 65 years

**Supplementary Figure S1.** ^18^F-florzolotau signals in the choroid plexus and adjacent brain regions in healthy individuals undergoing a dynamic PET scan

**Supplementary References**

**Supplementary Methods S1.**

**MRI data acquisition and scan parameters, and data processing**

Structural MRI scans were performed using a 3.0-T MRI scanner (MAGNETOM Verio, Siemens, Germany) for all participants, except for one patient who underwent scanning with a 1.5-T system. T1-weighted images were obtained using a 3D gradient-echo sequence, yielding a gapless series of thin sagittal sections with the following parameters: repetition time (TR) = 2,300 ms, echo time (TE) = 1.95 ms, inversion time (TI) = 900 ms, flip angle = 9°, field of view (FOV) = 250 mm, acquisition matrix = 256 × 256, and slice thickness = 1 mm.

Surface-based cortical reconstruction and volumetric subcortical segmentation were performed for each T1-weighted image using FreeSurfer software (version 7.3.2; <http://surfer.nmr.harvard.edu>).

**Supplementary Methods S2.**

**Details of PET scanners and acquisition protocols**

1) ^11^C-PiB PET

A 20-min steady-state PET scan (4×5-min frames) was performed beginning 50 min after the intravenous bolus injection of ^11^C-PiB. Scans were performed using one of the following PET systems.

i) A Biograph mCT flow system (Siemens Healthcare, Erlangen, Germany) was used for 26 patients and 38 controls. This system provides 109 axial slices with a 21.8-cm axial FOV and an intrinsic spatial resolution of 5.9 mm (in-plane) and 5.5 mm (axial) full-width at half-maximum (FWHM). Images were reconstructed using a filtered back-projection (FBP) algorithm with a Hanning filter (4.0 mm FWHM). Attenuation correction was performed using CT-based methods, and random coincidence correction was applied using the late coincidence counting method.
ii) The Siemens ECAT Exact HR+ system (CTI/Siemens, Knoxville, TN, USA) was used for five patients and three controls. This scanner provides 63 slices with a 15.5-cm axial FOV and 2.46-mm contiguous slice thickness, with transaxial and axial resolutions of 5.6 mm and 5.4 mm, respectively. Image reconstruction was performed with FBP using a Hanning filter (cutoff frequency: 0.4 cycles/pixel). Attenuation correction was based on a 10-minute transmission scan using a ^68^Ge/^68^Ga line source.
iii) Discovery MI (GE Healthcare, Chicago, Illinois, USA) was used in six patients and six controls. This system provides 89 axial slices with a 25-cm axial FOV and intrinsic spatial resolution of 4.8 mm (in-plane) and 5.3 mm (axial) FWHM. Images were reconstructed with FBP and a Hanning filter (4.0 mm FWHM). Attenuation correction was based on CT, and random correction was performed using a single-based estimation method.

2) ^18^F-florzolotau PET

Ninety minutes after an intravenous bolus injection of ^18^F-florzolotau, the participants underwent a 20-min PET acquisition (4×5-min frames) using the Biograph mCT flow system. To prevent photoracemization of the radiotracer, all injections and scans were performed in a dimly lit room with green or red lighting. The acquisition parameters were the same as those described for ^11^C-PiB PET using the Biograph system.

All PET images were corrected for scattering using a single-scatter simulation method. A head-fixation device was used to minimize head motion. To correct for head motion, the last three frames were realigned to the first 5-min frame using PMOD^®^ software (ver. 3.8; PMOD Technologies Ltd., Zurich, Switzerland). The quality of motion correction was verified by visual inspection.

**Supplementary Methods S3.**

**Details of ROI definition**

In this study, we utilized two anatomical atlases implemented in the FreeSurfer software ([1, 2]). The cerebellar gray matter (GM) ROI was defined using the atlas by Fischl et al. [1] and was employed as the reference region for quantifying ^11^C-PiB PET SUVR images. For the regional analysis of ^18^F-florzolotau SUVRs, the following GM ROIs were defined based on the atlas by Klein et al. [2]:

Frontal ROI: superior frontal gyrus, rostral middle frontal gyrus, caudal middle frontal gyrus, pars opercularis, pars triangularis, pars orbitalis, lateral orbitofrontal gyrus, medial orbitofrontal gyrus, precentral gyrus, and paracentral lobule

Temporal ROI: superior temporal gyrus, middle temporal gyrus, inferior temporal gyrus, transverse temporal gyrus, entorhinal cortex, parahippocampal gyrus, and fusiform gyrus

Parietal ROI: postcentral gyrus, supramarginal gyrus, superior parietal lobule, inferior parietal lobule, and precuneus

Occipital ROI: lingual gyrus, pericalcarine cortex, cuneus cortex, and lateral occipital cortex

**Supplementary Methods S4.**

**Individual evaluation of ^18^F-florzolotau images and tau positivity assessments in patients**

To examine tau positivity and tau distribution patterns in patients with late-onset psychosis (LOP) in more detail, we performed voxel-based analyses of parametric ^18^F-florzolotau standardized uptake value ratio (SUVR) images of each patient in comparison with the 46 amyloid-negative controls (one control with amyloid positivity by ^11^C-PiB PET was excluded) using Statistical Parametric Mapping software (SPM12; Wellcome Department of Imaging Neuroscience, London, UK). For the image preprocessing, MRI data of each participant were processed using SPM12 software and the CAT12 toolbox (http://www. neuro.uni-jena.de/cat) running on MATLAB R2018b (MathWorks, MA, USA). The segmented MRI and ^18^F-florzolotau SUVR images of all participants were then spatially normalized to the standardized Montreal Neurological Institute (MNI) 152 brain template. The normalized SUVR images were smoothed using a Gaussian kernel with an 8 mm full-width at half-maximum.

Two-sample t-tests were performed in SPM12 to compare the normalized SUVR data of each patient with those of the 46 healthy participants without amyloid positivity. In line with our previous study [3], the statistical threshold was set at *P* < .001 at the peak level, uncorrected, with an extent threshold of 30 voxels for the evaluation of tau positivity in the GM regions of each patient. Accumulation in areas adjacent to the choroid plexus, ventricles, or areas outside the brain parenchyma was not considered positive [4]. The topological pattern of tau-positive areas was subsequently examined (tau positivity in the GM regions of amyloid-negative controls has been previously reported, with six and 40 being tau-positive and tau-negative, respectively).

**Supplementary Methods S5.**

**Group differences in amyloid and tau accumulation after adjustment for educational attainment**

Considering the potential influence of educational attainment on amyloid and tau pathology [5-7], supplementary analyses were performed to evaluate group differences in amyloid and tau accumulation while adjusting for years of education.

*Amyloid and tau positivity*

First, logistic regression analyses were performed to examine group differences in amyloid and tau positivity rates, with educational level (years) included as a covariate. Statistical significance was set at *P* < .05.

*Regional ^18^F-florzolotau retention*

Subsequently, consistent with the primary analyses, a multivariate analysis of covariance (MANCOVA) was conducted to evaluate the effects of diagnosis (patients = 1, controls = 0) on ^18^F-florzolotau SUVR across four cortical regions (frontal, temporal, parietal, and occipital GM), with age and sex included as covariates. A statistical threshold of *P* < .05 was applied for the overall effect of diagnosis. When a significant main effect of diagnosis was observed, post-hoc analyses were performed to assess the SUVR differences in each ROI, with Bonferroni correction applied for the four ROIs (*P* < .05/4). The same analysis was performed in the Aβ-negative subgroup.

**Supplementary Methods S6.**

**Sensitivity analysis excluding healthy male controls younger than 65 years**

We performed a sensitivity analysis to examine group differences between patients and controls with respect to amyloid and tau positivity, as well as regional tau accumulation, after excluding healthy male controls younger than 65 years of age. This exclusion was intended to reduce the imbalance in the sex ratio (male: 27.0% [10/37] in patients vs. 53.2% [25/47] in controls) and the numerical difference in mean age (69.9 years in patients vs. 66.4 years in controls), given that the original patient cohort included only one male participant younger than 65 years of age (58 years).

Consistent with the primary analyses, chi-square tests were performed to investigate group differences in amyloid and tau positivity rates, with statistical significance defined as *P* < .05.

Subsequently, a MANCOVA was conducted to evaluate the effects of diagnosis (patients = 1, controls = 0) on ^18^F-florzolotau SUVR across four cortical regions (frontal, temporal, parietal, and occipital GM), with age and sex included as covariates. A statistical threshold of *P* < .05 was applied for the main effect of diagnosis. When a significant main effect was observed, post-hoc analyses were performed to compare the SUVRs in each ROI using the Bonferroni correction for the four ROIs (*P* < .05/4). The same analysis was repeated for the Aβ-negative subgroup.

**Supplementary Methods S7.**

**Relationship between ^18^F-florzolotau SUVR and the presence or history of clinical features related to psychosis**

We investigated the association between putative tau pathology and the presence or history of 1) partition delusions, 2) persecutory delusions, both of which are characteristic of LOP [8], and 3) hallucinations. None of the patients had a history of visual hallucinations.

First, for all patients, multivariate analysis of covariance (MANCOVA) was performed to separately examine the effects of each of these three clinical features on the ^18^F-florzolotau SUVR. The dependent variables were the SUVR values of the four ROIs (frontal, temporal, parietal, and occipital). The fixed factor was the presence or absence of each clinical feature, coded as a dummy variable (presence = 1, absence = 0). Age and sex were included as nuisance covariates. Statistical significance was set at *P* < .05.

Subsequently, we performed MANCOVA exclusively for patients who were Aβ-negative.

**Supplementary Results S1.**

**Analysis of group differences in amyloid and tau accumulation after adjustment for educational attainment**

*Amyloid and tau positivity*

Logistic regression analyses adjusted for educational attainment revealed that the patient group had significantly higher odds of both Aβ and tau positivity than the control group (Aβ: *P* = .005, odds ratio [OR] = 21.5, 95% confidence interval [CI], 2.5–186.9; tau: *P* < .001, OR = 9.67, 95% CI, 3.1–30.6).

*Regional ^18^F-florzolotau retention*

MANCOVA demonstrated a significant main effect of diagnosis on ^18^F-florzolotau SUVRs (*P* = .003). Subsequent post-hoc univariate analyses revealed significantly higher SUVRs in the parietal ROI in patients than in controls (*P* < .001, partial η^2^ = 0.13, power = 0.92), along with trend-level increases observed in the frontal (*P* = .013, partial η^2^ = 0.08, power = 0.71) and occipital (*P* = .020, partial η^2^ = 0.07, power = 0.65) ROIs.

When the analysis was restricted to Aβ-negative participants, the diagnostic effect on regional SUVRs remained significant (MANCOVA, *P* = .005, partial η ^2^ = 0.21, power = 0.90). Follow-up univariate analyses revealed a significantly higher SUVR in the parietal ROI (*P* = .004, partial η^2^ = 0.12, power = 0.83), along with a trend-level increase in the occipital ROI (*P* = .026, partial η^2^ = 0.07, power = 0.61) in Aβ-negative patients compared with Aβ-negative controls.

Overall, these findings are largely consistent with our primary analyses without adjusting for educational attainment. The only notable difference was that in the Aβ-negative subgroup analysis, the increase in occipital SUVR in patients was attenuated from statistical significance to a trend-level effect after controlling for education.

**Supplementary Results S2.**

**Sensitivity analysis excluding healthy male controls younger than 65 years**

Following the exclusion of 13 healthy male controls younger than 65 years of age, no significant differences or trend-level differences were observed between the groups with respect to age (*T* = 0.4, *P* = .71) or sex ratio (*χ^2^* = 0.6, *P* = .45) (Supplementary Table S1A).

*Amyloid and tau positivity*

In the adjusted control group (n = 34), amyloid and tau positivity was identified in one (2.9%) and seven (20.6%) participants, respectively. The rates of both amyloid and tau positivity were significantly higher in the patient group than in the control group (amyloid, *χ^2^* = 11.6, *P* < .001, Cramer’s *V* = 0.40; tau: *χ^2^* = 14.1, *P* < .001, Cramer’s *V* = 0.45).

*Regional ^18^F-florzolotau retention*

MANCOVA demonstrated a significant main effect of diagnosis on ^18^F-florzolotau SUVRs across cortical regions (*P* = .014). Subsequent post-hoc univariate analyses revealed significantly higher SUVRs in the parietal ROI in patients than in controls (*P* = .003), along with trend-level increases in the frontal (*P* = .020) and occipital (*P* = .047) ROIs (Supplementary Table S1B).

When the analysis was restricted to Aβ-negative participants, the effect of diagnosis on SUVRs remained significant (MANCOVA, *P* = .007). Follow-up univariate analyses demonstrated significantly higher SUVRs in the parietal ROI (*P* = .011, partial η^2^ = 0.12, power = 0.74), along with a trend-level increase in the occipital ROI (*P* = .028) in Aβ-negative patients compared with Aβ-negative controls (Supplementary Table S1C).

Overall, these results were largely consistent with the primary analyses. The only notable difference was observed in the post-hoc analysis restricted to the Aβ-negative participants, in which the increase in occipital SUVR in patients reached trend-level significance rather than statistical significance, possibly reflecting the reduced statistical power associated with the smaller sample size.

**Supplementary Results S3.**

**Relationship between ^18^F-florzolotau SUVR and the presence or history of clinical features related to psychosis**

For both analyses on all patients and exclusively on Aβ-negative patients, MANCOVA demonstrated no significant effects of the presence or history of either 1) partition delusion, 2) persecutory delusion, or 3) hallucination on the ^18^F-florzolotau SUVRs.
**Supplementary Discussion S1.**

**Potential spillover effects from ^18^F-florzolotau accumulation in the choroid plexus**

Accumulation of ^18^F-florzolotau radioactivity in the choroid plexus may potentially interfere with the quantitative assessment of tau deposition in adjacent brain structures, particularly the hippocampus. To investigate this possibility, we analyzed dynamic PET imaging data obtained from additional healthy control subjects (n = 4) included in our previous kinetic study [4]. As shown in Supplementary Figure S1, modest spillover effects from the choroid plexus were observed in the hippocampus and thalamus, whereas such effects appeared to be minimal in the entorhinal cortex and parahippocampal gyrus.

Furthermore, our recent clinicopathological study in a patient with progressive supranuclear palsy provided additional evidence regarding regional spillover effects (see Figure 7 of ref. [9]). In brain regions located immediately adjacent to the choroid plexus, such as the hippocampal CA4 and dentate gyrus, the ^18^F-florzolotau SUVR values tended to deviate upward from the regression line plotted against the AT8-positive tau load, suggesting possible contamination by spillover radioactivity. In contrast, the entorhinal cortex did not show a disproportionate increase in SUVR relative to the regression line.

Collectively, these findings indicate that spillover effects from choroid plexus ^18^F-florzolotau accumulation are limited in the entorhinal and parahippocampal regions, supporting the reliability of tau quantification in these regions.

**Supplementary Discussion S2.**

**Methodological considerations regarding the optimized reference tissue method**

Given that LOP may involve heterogeneous underlying pathologies, including frontotemporal lobar degeneration (FTLD), in which tau pathology can extend to the cerebellum, we employed an optimized reference tissue method rather than a conventional cerebellar GM ROI. This approach, consistent with our previous study on late-onset mood disorders [3], identifies reference voxels through frequency histogram analysis to minimize the potential influence of cerebellar tau accumulation.

For the calculation of ^18^F-florzolotau SUVRs, we applied the optimized reference tissue method using PET data acquired during the 90–110 min post-injection period, as previously described [10]. Briefly, reference voxels were identified by fitting a bimodal Gaussian distribution to the frequency histogram of the radioligand retention values within the GM tissue. The lower-intensity peak was assumed to represent tau-free tissue, and voxels within the full-width at half-maximum (FWHM) of this first peak were extracted and pooled to generate the reference region for SUVR estimation.

In the current cohort, the frequency histograms generally exhibited stable bimodal Gaussian distributions, indicating a relatively clear separation between tau-positive and tau-negative voxels, or monomodal distributions, suggesting minimal tau-positive voxel involvement. No case showed highly complex multimodal peaks. These observations support the robustness and stability of the present quantification approach for this dataset. Nonetheless, given the potential neuropathological heterogeneity underlying LOP, it remains possible that larger cohorts could include cases with more complex tracer retention distributions. In such situations, alternative approaches, including trimodal fitting models, may further reduce the potential quantification biases associated with bimodal fitting procedures.

**Supplementary Table S1. Group comparisons after excluding healthy male controls younger than 65 years**

(A) Group difference in demographic and experimental profiles

|  | Patients (N = 37) | |  | Controls (n = 34) | |  | Statistics | |
| --- | --- | --- | --- | --- | --- | --- | --- | --- |
|  | n | Mean (SD) |  | n | Mean (SD) |  | *T/chi-square* | *P* |
| Age, mean (SD), y | 37 | 69.9 (9.0) |  | 34 | 69.1 (8.5) |  | 0.4 | .71 |
| Sex (male/female) | 37 | 10/27 |  | 34 | 12/22 |  | 0.6 | .45 |
|  |  |  |  |  |  |  |  |  |
| Education level, mean (SD), y | 37 | 11.9 (3.3) |  | 34 | 14.6 (1.9) |  | -4.3 | <.001 ^3)^ |
| MMSE, mean (SD) ^1)^ | 36 | 25.1 (3.8) |  | 34 | 29.4 (0.7) |  | -6.6 | <.001 ^3)^ |
| FAB, mean (SD) ^2)^ | 34 | 12.7 (3.6) |  | 34 | 16.4 (1.3) |  | -5.6 | <.001 ^3)^ |
|  |  |  |  |  |  |  |  |  |
| Injected radioactivity for ^11^C-PiB, mean (SD), MBq | 37 | 541.8 (22.0) |  | 34 | 534.2 (43.6) |  | 0.9 | .35 |
| Injected radioactivity for ^18^F-florzolotau, mean (SD), MBq | 37 | 186.7 (8.3) |  | 34 | 186.4 (6.8) |  | 0.2 | .85 |

(B) Group differences in ^18^F-florzolotau SUVR including both amyloid positive and negative participants

|  | Patients (n = 37) |  | Controls (n = 34) |  | Multivariate tests (effect of diagnosis) | | |  | Between-subjects effects | | | |
| --- | --- | --- | --- | --- | --- | --- | --- | --- | --- | --- | --- | --- |
| SUVR | Mean (SD) |  | Mean (SD) |  | *F*(4,64) | *P* | Partial η^2^/  Power |  | *F*(1,67) | *P* | Partial η^2^ | Power |
|  |  |  |  |  |  |  |  |  |  |  |  |  |
|  |  |  |  |  | 3.38 | .014 ^3)^ | 0.17/  0.82 |  |  |  |  |  |
| Frontal | 0.92 (0.08) |  | 0.88 (0.04) |  |  |  |  |  | 5.72 | .020 | 0.08 | 0.66 |
| Temporal | 1.00 (0.09) |  | 0.99 (0.07) |  |  |  |  |  | 0.12 | .73 | 0.002 | 0.06 |
| Parietal | 0.97 (0.06) |  | 0.92 (0.08) |  |  |  |  |  | 9.39 | .003 ^4)^ | 0.12 | 0.86 |
| Occipital | 1.02 (0.09) |  | 0.98 (0.10) |  |  |  |  |  | 4.11 | .047 | 0.06 | 0.52 |

(C) Group differences in ^18^F-florzolotau SUVR in participants with amyloid-negative participants only

|  | Patients (n = 24) |  | Controls (n = 33) |  | Multivariate tests (effect of diagnosis) | | |  | Between-subjects effects | | | |
| --- | --- | --- | --- | --- | --- | --- | --- | --- | --- | --- | --- | --- |
| SUVR | Mean (SD) |  | Mean (SD) |  | *F*(4,50) | *P* | Partial η^2^/  Power |  | *F*(1,53) | *P* | Partial η^2^ | Power |
|  |  |  |  |  |  |  |  |  |  |  |  |  |
|  |  |  |  |  | 3.94 | .007 ^3)^ | 0.24/  0.88 |  |  |  |  |  |
| Frontal | 0.90 (0.05) |  | 0.88 (0.04) |  |  |  |  |  | 1.08 | .30 | 0.02 | 0.17 |
| Temporal | 0.97 (0.04) |  | 0.97 (0.04) |  |  |  |  |  | 1.44 | .24 | 0.03 | 0.22 |
| Parietal | 0.95 (0.04) |  | 0.91 (0.06) |  |  |  |  |  | 6.99 | .011 ^4)^ | 0.12 | 0.74 |
| Occipital | 1.03 (0.08) |  | 0.97 (0.10) |  |  |  |  |  | 5.10 | .028 | 0.09 | 0.60 |

Abbreviations: SUVR, standardized uptake value ratio

1) Data are unavailable for one patient

2) Data are unavailable for three patients

3) *P* < .05

4) *P* < .05/4 (post-hoc between-subjects effects)

**Supplementary Figure S1.**

**^18^F-florzolotau signals in the choroid plexus and adjacent brain regions in healthy individuals undergoing a dynamic PET scan**

(A) Representative ^18^F-florzolotau SUV images in the choroid plexus and adjacent ROIs (hippocampus, thalamus, entorhinal cortex, and parahippocampal gyrus) overlaid on T1-weighted MR images. PET data acquired from the 90–150 min post-injection period were used.

(B) Time-activity curves expressed as tissue-to-cerebellar GM radioactivity ratios for the choroid plexus and adjacent regions (n = 4).

The data were obtained from healthy individuals who participated in our previous study [4].

Abbreviations: GM, gray matter; ROI, region of interest; SUV, standardized uptake value

**Supplementary References**

1.	Klein A, Tourville J. 101 labeled brain images and a consistent human cortical labeling protocol. *Front Neurosci* 2012; **6:** 171.

2.	Fischl B, Salat DH, Busa E, Albert M, Dieterich M, Haselgrove C *et al.* Whole brain segmentation: Automated labeling of neuroanatomical structures in the human brain. *Neuron* 2002; **33**(3)**:** 341-355.

3.	Kurose S, Moriguchi S, Kubota M, Tagai K, Momota Y, Ichihashi M *et al.* Diverse tau pathologies in late-life mood disorders revealed by PET and autopsy assays. *Alzheimers Dement* 2025; **21**(6)**:** e70195.

4.	Tagai K, Ono M, Kubota M, Kitamura S, Takahata K, Seki C *et al.* High-contrast *in vivo* imaging of tau pathologies in Alzheimer's and non-Alzheimer's disease tauopathies. *Neuron* 2021; **109**(1)**:** 42-58 e48.

5.	Cai Y, Fang L, Li A, Yang J, Zhou X, He Z *et al.* Educational attainment, Aβ, tau, and structural brain reserve in Alzheimer's disease. *Alzheimers Dement* 2025; **21**(2)**:** e14400.

6.	Pichet Binette A, Vachon-Presseau E, Morris J, Bateman R, Benzinger T, Collins DL *et al.* Amyloid and tau pathology associations with personality traits, neuropsychiatric symptoms, and cognitive lifestyle in the preclinical phases of sporadic and autosomal dominant Alzheimer's disease. *Biol Psychiatry* 2021; **89**(8)**:** 776-785.

7.	Ossenkoppele R, Lyoo CH, Jester-Broms J, Sudre CH, Cho H, Ryu YH *et al.* Assessment of demographic, genetic, and imaging variables associated with brain resilience and cognitive resilience to pathological tau in patients with Alzheimer disease. *JAMA Neurol* 2020; **77**(5)**:** 632-642.

8.	Howard R, Rabins PV, Seeman MV, Jeste DV. Late-onset schizophrenia and very-late-onset schizophrenia-like psychosis: An international consensus. The international late-onset schizophrenia group. *Am J Psychiatry* 2000; **157**(2)**:** 172-178.

9.	Araki N, Takeda T, Takado Y, Tagai K, Seki E, Arai N *et al.* Florzolotau (18F) PET provides in-vivo measures of tau pathology in progressive supranuclear palsy: An imaging-postmortem correlation study. *medRxiv* 2025.

10.	Tagai K, Ikoma Y, Endo H, Debnath OB, Seki C, Matsuoka K *et al.* An optimized reference tissue method for quantification of tau protein depositions in diverse neurodegenerative disorders by PET with ^18^F-PM-PBB3 (^18^F-APN-1607). *Neuroimage* 2022; **264:** 119763.
